# Strong Chang's Conjecture and the tree property at *ω*_2_^☆^

**DOI:** 10.1016/j.topol.2015.05.061

**Published:** 2015-12

**Authors:** Víctor Torres-Pérez, Liuzhen Wu

**Affiliations:** a Institut für Diskrete Mathematik und Geometrie, TU Wien, Wiedner Haupstraße 8-10/104, 1040 Vienna, Austria; b Institute of Mathematics, Chinese Academy of Sciences, East Zhong Guan Cun Road No. 55, Beijing 100190, China

**Keywords:** Strong Chang's Conjecture, Tree property

## Abstract

We prove that a strong version of Chang's Conjecture together with $2^{\omega }=\omega_{2}$ implies there are no $\omega_{2}$-Aronszajn trees.

## 1 Introduction

Given a regular cardinal *κ*, we say that *κ* has the *tree property* if every tree *T* of height *κ* and levels of size <*κ*, *T* has a cofinal branch, and it is usually denoted by TP(κ). Trees of height *κ* with levels of size <*κ* with no cofinal branches are usually called *κ*-*Aronszajn*.

We list some historical results involving the Tree Property for different regular cardinals. König's Lemma gives some sufficient conditions for a tree to have a cofinal branch. He proved in [5] that TP(ω) holds. However, Aronszajn showed that we cannot generalize König's Lemma for trees of height $\omega_{1}$ by constructing an $\omega_{1}$-Aronszajn tree (see [2]). Considering trees of height $\omega_{2}$ with levels of size at most ${ℵ}_{1}$, it turns out to be independent from the usual axioms of Set Theory. We recall also the result by Silver, where if $\mathrm{TP}(\omega_{2})$ holds, then ${ℵ}_{2}$ is weakly compact in L (Theorem 5.9 in [6]). On the other hand, Mitchell proved that if *κ* is a weakly compact, then there is a generic extension where $\kappa =\omega_{2}=2^{\omega }$ and $\mathrm{TP}(\omega_{2})$ holds (see [6]). In particular, $\mathrm{TP}(\omega_{2})$ is equiconsistent with the existence of a weakly compact cardinal.

In these notes we work with a strong version of Chang's Conjecture (see Definition 3.1 of the present notes and also see Theorem 1.3 in [9] for an earlier reference) here denoted by ${\mathrm{CC}}^{⁎}$. On one hand, Todorčević and Torres-Pérez proved that under a stronger version of ${\mathrm{CC}}^{⁎}$, the negation of CH implies there are no special $\omega_{2}$-Aronszajn trees (see [15]). On the other hand, Sakai and Velickovic proved that under SSR, a strengthening of ${\mathrm{CC}}^{⁎}$ (see [1]), the negation of CH together with ${\mathrm{MA}}_{\omega_{1}}(\mathrm{Cohen})$ implies the strong tree property at $\omega_{2}$ and so in particular it implies $\mathrm{TP}(\omega_{2})$ (see [8]).

We prove in these notes that ${\mathrm{CC}}^{⁎}$ and the negation of CH imply $\mathrm{TP}(\omega_{2})$. Observe that by a result of Todorčević (see [14]), ${\mathrm{CC}}^{⁎}$ implies $2^{\omega }\leq \omega_{2}$, so under ${\mathrm{CC}}^{⁎}$, ¬CH is equivalent to $2^{\omega }=\omega_{2}$.

We make a remark for the necessity of $\neg {\mathrm{MA}}_{\omega_{1}}(\mathrm{Cohen})$ in [8]:

Theorem 1.1 **Folklore**

*Assume that there exists a strongly compact cardinal. Then there exists a forcing extension in which* $\mathrm{SSR}+\neg {\mathrm{MA}}_{\omega_{1}}(\mathrm{Cohen})+\neg \mathrm{CH}$ *holds.*

The following fact is used:

Fact 1.1 *(Shelah* *[10]**, Chapter XIII, 1.6 and 1.10) Assume that κ is a strongly compact cardinal. Let* $\left(P_{\alpha },{\overset{˙}{Q}}_{\beta }:\alpha \leq \kappa ,\beta <\kappa \right)$ *be a revised countable support iteration of semi-proper posets of size* <*κ such that* $\kappa =\omega_{2}$ *in* $V^{P_{\kappa }}$*. Then* SSR *holds in* $V^{P_{\kappa }}$*.*

Assume that *κ* is strongly compact in *V*. Let $\left(P_{\alpha },{\overset{˙}{Q}}_{\beta }:\alpha \leq \kappa ,\beta <\kappa \right)$ be the countable support iteration of random forcing. Here recall that a revised countable support iteration coincides with a countable support iteration for proper posets. Note also that $\kappa =\omega_{2}$ in $V^{P_{\kappa }}$. Hence SSR holds in $V^{P_{\kappa }}$ by the above fact. Moreover, ${\mathrm{MA}}_{\omega_{1}}(\mathrm{Cohen})$ fails in $V^{P_{\kappa }}$ as adding random reals makes non(B) into $\omega_{1}$.

## 2 Preliminaries and basic definitions

Given a limit ordinal *γ*, a subset A⊆γ is *unbounded in γ* if sup⁡(A)=γ. *A* is *closed* in *γ* if for every limit ordinal β<γ, if A∩β is unbounded in *β*, then β∈A. A set A⊆γ is often called a *club set in γ* if it is closed and unbounded in *γ*. A set S⊆γ is stationary, if S∩A≠∅ for every *A* club in *γ*.

The following result involving stationary sets is known as *Fodor's Lemma* or the *Pressing Down Lemma for ordinals*.

Lemma 2.1 *(Fodor* *[3]**) Let κ be a regular uncountable cardinal. Then for every* S⊆κ *stationary, and for every* f:S→κ *such that* f(α)<α *for every* α∈S*, there is* ξ<κ *such that* $f^{-1}(\{\xi \})$ *is stationary.*

We use a general version of a stationary set, originally by Jech, but in these notes we use an equivalent version due to Kueker (see for example, Theorem 8.28 in [4]). Given an infinite set *A*, we denote by ${\left[A\right]}^{<\omega }$ the collection of finite subsets of *A*. Similarly, let ${\left[A\right]}^{\omega }$ denote the collection of all subsets of *A* of size *ω*. We say that a set $S\subseteq {\left[A\right]}^{\omega }$ is *stationary in* ${\left[A\right]}^{\omega }$ if for every function $F:{\left[A\right]}^{<\omega }\to A$, there is $X\in {\left[A\right]}^{\omega }$ such that F(e)∈X for every $e\in {\left[X\right]}^{<\omega }$.

The following lemma is the generalized version of the Pressing Down Lemma (see Theorem 8.24 in [4]).

Lemma 2.2 **Jech**

*For every stationary set* $S\subseteq {\left[A\right]}^{\omega }$ *and for every function* f:S→A *such that* f(X)∈X *for every* X∈S*, there is* a∈A *such that* $f^{-1}(\{a\})$ *is stationary.*

The couple $\langle T,{<}_{T}\rangle$ is a *tree* whenever ${<}_{T}$ is a partial order of *T*, and for every t∈T, the set $\left\{s\in T:s{<}_{T}t\right\}$ is well-ordered by ${<}_{T}$. Some times we may just write the tree *T*, assuming there is an implicit order. We denote by ${\mathrm{pred}}_{T}(t)$ the set of all the ${<}_{T}$-predecessors of *t* in *T*, and by ${\mathrm{ht}}_{T}(t)=o.t.({\mathrm{pred}}_{T}(t))$. We will denote by $T_{\xi }=\{t\in T:{\mathrm{ht}}_{T}(t)=\xi \}$. Often we will just drop off the subindex *T* if the context is clear.

For A,B⊆T we denote by A⊥B if for every s∈A and every t∈B, *s* and *t* are not comparable. Similarly, for s,t∈T and A⊆T, let s⊥t and s⊥A iff {s}⊥{t} and {t}⊥A respectively.

Given an ordinal $\lambda \geq \omega_{2}$, we recall the *Weak Reflection Principle for λ*, WRP(λ).

Definition 2.1 WRP(λ) is the following statement: For any stationary subset *S* of ${\left[\lambda \right]}^{\omega }$, there is X⊂λ such that

(1) $|X|=\omega_{1}$,
(2) $\omega_{1}\subseteq X$ and $S\cap {\left[X\right]}^{\omega }$ is a stationary subset of ${\left[X\right]}^{\omega }$.

Todorčević showed the following (see Lemma 6 in [14]):

Lemma 2.3 **Todorčević**

${\mathrm{CC}}^{⁎}$ *implies* $\mathrm{WRP}(\omega_{2})$*.*

## 3 Main Theorem

In this section we prove our main result.

Theorem 3.1 *Under* ${\mathrm{CC}}^{⁎}$*,* ¬CH *is equivalent to the tree property at* $\omega_{2}$*.*

We follow very closely the proof of Theorem 2.2 in [15]. It is a classical result of Specker that $\mathrm{TP}(\omega_{2})$ implies ¬CH (see [11]).

Given two sets $M^{⁎},M$ we will denote by $M^{⁎}⊒M$ iff $M^{⁎}⊇M$ and $M^{⁎}\cap \omega_{1}=M\cap \omega_{1}$. Consider the following strong version of Chang's Conjecture:

Definition 3.1 **${\mathrm{CC}}^{⁎}$**

There are arbitrarily large uncountable regular cardinals *θ* such that for every well-ordering < of $H_{\theta }$, and every countable elementary submodel $M≺\langle H_{\theta };\in ,<\rangle$, and every ordinal $\eta <\omega_{2}$, there exists an elementary countable submodel $M^{⁎}≺\langle H_{\theta };\in ,<\rangle$ such that $M^{⁎}⊒M$ and $(M^{⁎}\cap \omega_{2})\setminus \eta \neq \emptyset$.

We will need the following Proposition for the proof of Lemma 3.1, namely in Claim 3.1.

Proposition 3.1 *Let T be a κ-Aronszajn tree (κ a regular cardinal). Given a regular cardinal* μ<κ*, consider a family of collection of nodes* $\langle A_{\xi }:\xi \in X\rangle$ *such that X contains a stationary set consisting of ordinals of cofinality at least μ,* $A_{\xi }\subseteq T_{\xi }$ *and* $|A_{\xi }|<\mu$ *for every* ξ∈X*. Then for every λ large enough such that* $\{\kappa ,T,X,\langle A_{\xi }:\xi \in X\rangle ,\ldots \}\subset H_{\lambda }$ *and for every elementary submodel* $N≺\langle H_{\lambda };\in ,<,\kappa ,T,X,\langle A_{\xi }:\xi \in X\rangle ,\ldots \rangle$ *such that* $A_{\xi }\subseteq N$ *for every* ξ∈X∩N*, then for every* t∈T *of height at least* sup⁡(N∩κ) *there are unboundedly many (in* sup⁡(N∩κ)*)* ξ∈X∩N *such that every* $s\in A_{\xi }$ *is incomparable with t.*

Proof Suppose otherwise, and take t∈T of height at least sup⁡(N∩κ) and α∈N such that for all ξ∈X∩N∖α, there is a node $t_{\xi }\in A_{\xi }$ such that $t_{\xi }\leq_{T}t$. Without loss of generality, we can suppose that *X* is a stationary set consisting of ordinals greater than *α* and of cofinality at least *μ*.

Since $|A_{\xi }|<\mu$ for any ξ∈X, there is an ordinal $\beta_{\xi }<\xi$ such that for any $s,s^{\prime }\in A_{\xi }$, $s=s^{\prime }↔s↾\beta_{\xi }=s^{\prime }↾\beta_{\xi }$. By elementarity and using Fodor's Lemma, we can find β∈N∩X and a stationary set S∈N such that for any ξ∈S, $s=s^{\prime }↔s↾\beta =s^{\prime }↾\beta$ for any $s,s^{\prime }\in A_{\xi }$.

Then for every $s\in A_{\beta }$, we can define a function $f_{s}:S\to T$ such that $f_{s}(\xi )$ is the unique $s_{\xi }\in A_{\xi }$ such that $s_{\xi }>s$. Since $A_{\beta }\subseteq N$, in particular s=t↾β∈N, and therefore $f_{s}$ is defined in *N*. However, by our initial assumption, $f_{s}(\xi )=t_{\xi }$ for every ξ∈S∩N, and so $f_{s}$ defines in *N* a cofinal branch of *T*, contradiction.  □

Let *T* be an $\omega_{2}$-Aronszajn tree. In order to simplify the proof, without loss of generality, we suppose that $T\subseteq \omega_{2}$ and let $e:\omega_{2}\times \omega_{1}\to T$ be a bijective function such that $e(\delta ,\xi )\in T_{\delta }$ for every $(\delta ,\xi )\in \omega_{2}\times \omega_{1}$. Let *θ* be sufficiently large such that *T*, *e* and all relevant parameters are members of $H_{\theta }$.

Lemma 3.1 *Assume* ${\mathrm{CC}}^{⁎}$ *and that T is an* $\omega_{2}$*-Aronszajn tree. For every* $M≺H_{\theta }$ *countable, and for every* $\eta_{0},\eta_{1}\in \omega_{2}$*, we can find* $M_{0},M_{1}≺H_{\theta }$ *countable such that:*

(1) $M\cap \omega_{1}=M_{0}\cap \omega_{1}=M_{1}\cap \omega_{1}$*,*
(2) $M_{0}\cap \omega_{2}\setminus \eta_{0}\neq \emptyset$ *and* $M_{1}\cap \omega_{2}\setminus \eta_{1}\neq \emptyset$*,*
(3) $\exists \delta_{0}\in (M_{0}\cap \omega_{2})$ *and* $\delta_{1}\in (M_{1}\cap \omega_{2})$ *such that* $\left(M_{0}\cap T_{\delta_{0}})⊥(M_{1}\cap T_{\delta_{1}}\right)$*.*

Proof Fix λ>θ sufficiently large such that ${\mathrm{CC}}^{⁎}$ holds in $H_{\lambda }$ and $M,\eta_{0},\eta_{1}$ and all relevant parameters are in $H_{\lambda }$. Let $N≺H_{\lambda }$ such that if $\gamma =\sup(N\cap \omega_{2})$, then $\mathrm{cof}(\gamma )=\omega_{1}$.

Fix $M_{1}$ witnessing CC* for *M* and *γ*.

We need the following Claim:

Claim 3.1 *For every* t∈T *of height at least γ, there is* $M^{⁎}⊒M$ *with* $M^{⁎}\in N$ *and* $\beta \in M^{⁎}\cap \omega_{2}$ *such that* $t⊥T_{\beta }\cap M^{⁎}$*.*

Proof Assume otherwise, and take t∈T of height at least *γ* such that for every $M^{⁎}\in N$ with $M^{⁎}⊒M$, for each $\beta \in M^{⁎}\cap \omega_{2}$, there is an $s_{\beta }\in (T_{\beta }\cap M^{⁎})$ such that $s_{\beta }<t$.

We work inside *N* in this paragraph. Using that ${\mathrm{CC}}^{⁎}$ holds in *N*, build a sequence of models $\langle M_{\eta }:\eta \in \omega_{2}\rangle$ such that $M_{\eta }⊒M$ and $M_{\eta }\cap \omega_{2}\setminus \eta \neq \emptyset$ for every $\eta \in \omega_{2}$. Let $\beta_{\xi }$ be the minimum $\beta \in \omega_{2}\setminus \xi$ such that there is $\eta \in \omega_{2}$ such that $\beta_{\xi }=\min(M_{\eta }\cap \omega_{2}\setminus \eta )$. Let $\eta_{\xi }$ be the minimum $\eta \in \omega_{2}$ such that $\beta_{\xi }=\min(M_{\eta }\cap \omega_{2}\setminus \eta )$. Define $\langle A_{\xi }:\xi \in \omega_{2}\rangle$ by setting $A_{\xi }$ to be the set of nodes *r* in $T_{\xi }$ with r≤s for some $s\in M_{\eta_{\xi }}\cap T_{\beta_{\xi }}$. Remark that since $M_{\eta_{\xi }}$ is countable, so is $A_{\xi }$.

By Proposition 3.1, there are unboundedly many $\xi \in N\cap \omega_{2}$ such that $t⊥A_{\xi }$, so choose one of such *ξ*'s. Then there is $s\in M_{\eta_{\xi }}\cap T_{\beta_{\xi }}$ such that $s{<}_{T}t$. Thus there is $r\in A_{\xi }$ such that $r\leq_{T}s{<}_{T}t$, contradicting that *r* and *t* are incomparable.  □

Let $\left\{t_{n}:n\in \omega \right\}$ be an enumeration of $M_{1}\cap T\setminus \gamma$. Using Claim 3.1, build a ⊆-increasing sequence $\langle M_{n}^{0}:n\in \omega \rangle$ of countable elementary submodels of $H_{\theta }$ such that for every n∈ω, $M_{n}^{0}\in N$ and $M_{n}^{0}⊒M$, and such that there is $\beta \in M_{n}^{0}\cap \omega_{2}$ with $t_{n}⊥M_{n}^{0}\cap T_{\beta }$. Let $M_{0}$ be an end-extension of ${⋃}_{n<\omega }M_{n}^{0}$ derived from ${\mathrm{CC}}^{⁎}$ and $\eta_{0}$. Let $\delta_{0}=\min(M_{0}\cap \omega_{2}\setminus \eta_{0})$ and $\delta_{1}=\min(M_{1}\cap \omega_{2}\setminus \gamma )$. We claim it suffices.

Take $s\in T_{\delta_{0}}\cap M_{0}$ and $t\in T_{\delta_{1}}\cap M_{1}$. In particular, there is n∈ω and $\beta \in M_{n}^{0}\cap \omega_{2}$ such that $t=t_{n}$ and $t⊥T_{\beta }\cap M_{n}^{0}$. Since $\beta \in M_{n}^{0}\subseteq M_{0}$, we have ${s↾}_{\beta }\in M_{0}$. Moreover, since the enumeration function $e\in M_{n}^{0}\subseteq M_{0}$ and $M_{n}^{0}\cap \omega_{1}=M_{0}\cap \omega_{1}$, we have $T_{\beta }\cap M_{0}=T_{\beta }\cap M_{n}^{0}$ and so ${s↾}_{\beta }\in M_{n}^{0}$. Therefore ${s↾}_{\beta }$ is not comparable with *t*, and so neither are *s* and *t*.

This finishes the proof of Lemma 3.1.  □

Lemma 3.2 *Assume* ${\mathrm{CC}}^{⁎}$*. Let T be an* $\omega_{2}$*-Aronszajn tree. If the set*

$$S_{T}=\{A\in {\left[\omega_{2}\right]}^{\omega }:\forall t\in T(\mathrm{pred}(t)\cap A\text{ is bounded in }\sup(A))\}$$

*is nonstationary, then* CH *holds.*

Proof Let $f:{\left[\omega_{2}\right]}^{<\omega }\to \omega_{2}$ such that the set $C_{f}$ of closure points of *f* (i.e. $X\in C_{f}$ iff for every $e\in {\left[X\right]}^{<\omega }$, f(e)∈X) is disjoint with $S_{T}$. We can suppose that $T\subseteq \omega_{2}$ and $e:\omega_{1}\times \omega_{2}\to T$ is a bijection such that $e(\delta ,\beta )\in T_{\delta }$. Let *λ* be sufficiently large such that *T*, $S_{T}$, *f*, *e* and all relevant parameters are members of $H_{\lambda }$.

Using previous lemma, build a binary tree ${\langle M_{\sigma }\rangle }_{\sigma \in 2^{<\omega }}$ of countable elementary submodels of $H_{\lambda }$ with the property that for every $\sigma \in 2^{<\omega }$

(1) $M_{\sigma }\cap \omega_{1}=M_{\sigma ⌢0}\cap \omega_{1}=M_{\sigma ⌢1}\cap \omega_{1}$,
(2) $M_{\sigma }\cap \omega_{2}⊊M_{\sigma ⌢0}\cap \omega_{2}$ and $M_{\sigma }\cap \omega_{2}⊊M_{\sigma ⌢1}\cap \omega_{2}$,
(3) there exists $\delta_{0}\in (M_{\sigma ⌢0}\cap \omega_{2})$ and $\delta_{1}\in (M_{\sigma ⌢1}\cap \omega_{2})$ such that $T_{\delta_{0}}\cap M_{\sigma ⌢0}⊥T_{\delta_{0}}\cap M_{\sigma ⌢1}$,
(4) for every $r\in 2^{\omega }$, if $M_{r}=\underset{n\in \omega }{⋃}M_{r↾n}$, then for every $r,r^{\prime }\in 2^{\omega }$, $\sup(M_{r}\cap \omega_{2})=\sup(M_{r^{\prime }}\cap \omega_{2})$.

Let *δ* be the common supremum of every $M_{r}\cap \omega_{2}$, $r\in 2^{\omega }$. Then for every $r\in 2^{\omega }$, there is $t_{r}\in T_{\delta }\cap M_{r}$ such that for every $\mathrm{pred}(t_{r})\cap M_{r}$ is unbounded in *δ*.

Claim 3.2 *The application* $r\mapsto t_{r}$ *is an injection from* $2^{\omega }$ *to* $T_{\delta }$ *(and so* CH *does hold).*

Proof Let $r_{0},r_{1}\in 2^{\omega }$ with $r_{0}\neq r_{1}$ and denote by $t_{i}$ the node $t_{r_{i}}$ for i∈{0,1}. We will find two predecessors of $t_{0}$ and $t_{1}$ that are incomparable.

Let n∈ω such that ${{r_{0}↾}_{n}=r_{1}↾}_{n}=\sigma$, and ${{r_{0}↾}_{n+1}\neq r_{1}↾}_{n+1}$. Without loss of generality suppose $r_{i}(n)=i$ for i∈{0,1}.

Since $(M_{r_{i}}\cap \omega_{2})\notin S_{T}$, we can find $s_{i}{<}_{T}t_{i}$ with $s_{i}\in M_{{r_{i}↾}_{m_{i}}}$ for some $m_{i}>n$. By the construction of our binary tree, we can take $\delta_{0}\in M_{{r_{0}↾}_{n+1}}$ and $\delta_{1}\in M_{{r_{1}↾}_{n+1}}$ such that $T_{\delta_{0}}\cap M_{{r_{0}↾}_{n+1}}⊥T_{\delta_{1}}\cap M_{{r_{1}↾}_{n+1}}$. However, observe that for i∈{0,1}, $\delta_{i}\in M_{{r_{i}↾}_{n+1}}\subseteq M_{r_{1}}$, and so ${t_{i}↾}_{\delta_{i}}\in M_{{r_{i}↾}_{n+1}}$. Therefore, ${t_{0}↾}_{\delta_{0}}$ and ${t_{1}↾}_{\delta_{1}}$ are incomparable, and so $t_{0}\neq t_{1}$.  □

This finishes the proof of Lemma 3.2.  □

We are now ready to finish the proof of our Theorem. From the previous lemma we know that the set $S_{T}$ is stationary in ${\left[\omega_{2}\right]}^{\omega_{0}}$. Let $S_{T}^{\prime }=S_{T}\cap C_{e}$, where $C_{e}$ is the club of all countable subsets of $\omega_{2}$ closed under the level enumeration function *e* of *T*.

We now use that ${\mathrm{CC}}^{⁎}$ implies $\mathrm{WRP}(\omega_{2})$ (Lemma 2.3). Take $X\subseteq \omega_{2}$ of size ${ℵ}_{1}$ such that $\omega_{1}\subseteq X$ and where $S_{T}^{\prime }\cap {\left[X\right]}^{\omega }$ is stationary. Take t∈T of height at least sup⁡(X).

From the definition of $S_{T}$, for every $A\in S_{T}^{\prime }\cap {\left[X\right]}^{\omega }$ we can choose $\beta_{A}\in A$ such that if s∈pred(T)∩A, then $s<\beta_{A}$. By the Pressing Down Lemma, there is a stationary set $S\subseteq S_{T}^{\prime }\cap {\left[X\right]}^{\omega }$ and a *β* such that $\beta_{A}=\beta$ for all A∈S. Let $\xi \in \omega_{1}$ such that ${e(\beta ,\xi )=t↾}_{\beta }$. Observe that *S* is in particular cofinal in ${\left[X\right]}^{\omega }$ so ⋃S=X. Since $\omega_{1}\subseteq X$, pick A∈S such that ξ∈A. Therefore, e(β,ξ)∈A∩pred(t), and so e(β,ξ)<β. But this is a contradiction, since in general e(β,ξ)≥β for any $\beta \in \omega_{2}$. This ends the proof of our Theorem.

## 4 Some final remarks

We mention some related previous results. R. Strullu proved that the Map Reflection Principle, introduced by Moore in [7], together with ${\mathrm{MA}}_{\omega_{1}}$ implies $\mathrm{TP}(\omega_{2})$ (see [12]). Also it is implicit in B. Velickovic and H. Sakai's results ([8]) that $\mathrm{WRP}(\omega_{2})+{\mathrm{MA}}_{\omega_{1}}(\mathrm{Cohen})$ implies $\mathrm{TP}(\omega_{2})$.

We remark that the results in [15] were in the context of Rado's Conjecture (RC), which is the following statement in Todorčević's equivalent version:

Definition 4.1 **RC**

Every tree *T* of height ${ℵ}_{1}$ is special, i.e., the countable union of antichains if and only if every subtree of *T* of size ${ℵ}_{1}$ is also special.

Todorčević proved via a large cardinal that RC is consistent, and showed it is independent from ZFC. In particular, RC is not compatible with ${\mathrm{MA}}_{\omega_{1}}$ (see final remarks in [13]).

As we have mentioned, in [15], it was proved that Rado's Conjecture together with the negation of the Continuum implies there are no special $\omega_{2}$-Aronszajn trees. One natural question was which extra condition we could add to Rado's Conjecture to obtain that there are no $\omega_{2}$-Aronszajn trees at all. Since Rado's Conjecture is consistent with both CH and ¬CH, and CH implies $\neg \mathrm{TP}(\omega_{2})$, we needed at least to add the condition ¬CH to RC if we wanted to obtain $\mathrm{TP}(\omega_{2})$. However, as we have mentioned, RC is not consistent with ${\mathrm{MA}}_{\omega_{1}}$, so we could not have similar results as the one cited above.

Todorčević proved in [14] that RC implies ${\mathrm{CC}}^{⁎}$. Therefore, a consequence of the result in the present paper is that the condition ¬CH was not only needed, but also sufficient to add to RC to get $\mathrm{TP}(\omega_{2})$.

Corollary 4.1 RC *and* ¬CH *imply* $\mathrm{TP}(\omega_{2})$*.*

As we have mentioned, Todorčević proved in [14] that ${\mathrm{CC}}^{⁎}$ implies $\mathrm{WRP}(\omega_{2})$. The following question is still open.

Question 4.1 *Do* $\mathrm{WRP}(\omega_{2})$ *and* ¬CH *imply together* $\mathrm{TP}(\omega_{2})$*?*
